# Galvanic current dosage and bacterial concentration are determinants of the bactericidal effect of percutaneous needle electrolysis: an in vitro study

**DOI:** 10.1038/s41598-021-98451-5

**Published:** 2021-09-23

**Authors:** José Antonio García-Vidal, Jesús Salinas, Pilar Escolar-Reina, Francisco Cuello, Nieves Ortega, Juan de Dios Berná-Mestre, Manuel López-Nicolás, Fermín Valera-Garrido, Francesc Medina-Mirapeix

**Affiliations:** 1 Department of Physiotherapy, University of Murcia, Campus de Espinardo, 30100 Murcia, Spain; 2 Department of Animal Health, Faculty of Veterinary, University of Murcia, Campus de Espinardo, 30100 Murcia, Spain; 3 Department of Radiology, Virgen de la Arrixaca University Clinical Hospital, El Palmar, 30120 Murcia, Spain; 4 Department of Dermatology, Stomatology, Radiology and Physical Medicine, University of Murcia, 30008 Murcia, Spain; 5 MVClinic Institute, Madrid, Spain

**Keywords:** Microbiology, Medical research, Therapeutics, Musculoskeletal system, Chronic inflammation

## Abstract

Percutaneous needle electrolysis (PNE) is a physiotherapy technique that has been shown to be effective in different pathologies such as tendinopathies or mammary fistula. For many years, theoretical bactericidal and germicidal effects have been attributed to this type of galvanic currents, partly explained by the changes in pH that it generates. However, these effects have not yet been demonstrated. The aim of this study was to evaluate the bactericidal effect and the changes in pH caused by PNE. *S. aureus* were prepared in two different solutions (TSB and saline solution) and in different concentrations (from 9 to 6 Log_10_ CFU/mL). Bacteria were treated with three experimental PNE doses to assess bacterial death levels and the changes caused to the pH of the medium. The viable cell count showed that all experimental PNE doses had a bactericidal effect against a high concentration (9 Log_10_ CFU/mL) of *S. aureus* in saline solution (p < 0.001). Furthermore, we found that when the concentration of bacteria decreased, a lower dose of galvanic current generated the same effect as a higher dose. Changes in pH were registered only in experiments performed with saline solution. PNE had a bactericidal effect against *S. aureus* and the level of this effect was mainly modulated by the solution, the bacterial concentration and the dose. Changes affecting pH were modulated by the type of solution and there was no relationship between this and bacterial death.

## Introduction

Percutaneous Needle Electrolysis (PNE) is a novel, minimally invasive approach involving the application of a galvanic electric current through a needle^1^. This procedure generates different alkaline molecules, capable of generating a non-thermal electrochemical ablation by cathodic flow directly into the affected tissue^2^.

Since the origins of this technique almost 20 years ago^3^, PNE has demonstrated clinical effectiveness on inflammatory areas associated with degenerative processes such as tendinopathies^4–6^. Bearing in mind that these areas are usually free of bacterial pathogens, PNE is theoretically applied on an aseptic medium. Any minimal trace of bacteria is most likely due to dragging through skin^7^ considering that this technique involves a percutaneous application. Traditionally, it has been speculated that the effectiveness in this medium could be due to changes in the pH induced around the needle^5^. Nevertheless, recently, an in vitro study has shown that discrete variations in body fluid composition may modulate pH changes^8^. Specifically, they have shown that the effect of the galvanic current on an aseptic saline solution generates greater pH changes than in Ringer solution.

In recent years, PNE has also proven to be clinically effective in cases of acute inflammation associated with bacterial infections by *Staphylococcus aureus* (*S. aureus*), such as mammary fistula (MF)^9,10^. It has also been speculated that the effectiveness of PNE within this bacterial solution could be facilitated by a bactericidal effect^9^. Nevertheless, although electrolysis is a physical phenomenon with a recognized disinfectant capacity against pathogens^11^ in different mediums and applications including industrial uses (food, swimming pools, wastewater…)^12–14^ or sanitary applications (medical supplies and surgical procedures)^15,16^, PNE has not yet demonstrated this capacity in neither in vitro nor in vivo studies. This paper attempts to provide new knowledge in this area using an in vitro design, as well as to extend the existing knowledge about the effect on changes in pH. For example, as the cited in vitro study^8^ only used one dose (3 mA-3 s-3 times) it is still unknown whether the effect of the type of solution on the pH changes also depends of the level of dose.

Considering the above, the main objective of our study was to evaluate the bactericidal efficacy of PNE and to establish whether there are patterns of interaction between the dose and different bacterial solutions or bacterial concentrations of the *S. aureus*. A secondary objective was to determine whether different bacterial solutions could also modulate the effect on the pH changes and whether this effect depends on the dosage level in vitro and on different parameters as the intensity and total dose.

## Materials and methods

### Bacteria preparation

**Figure 1:**
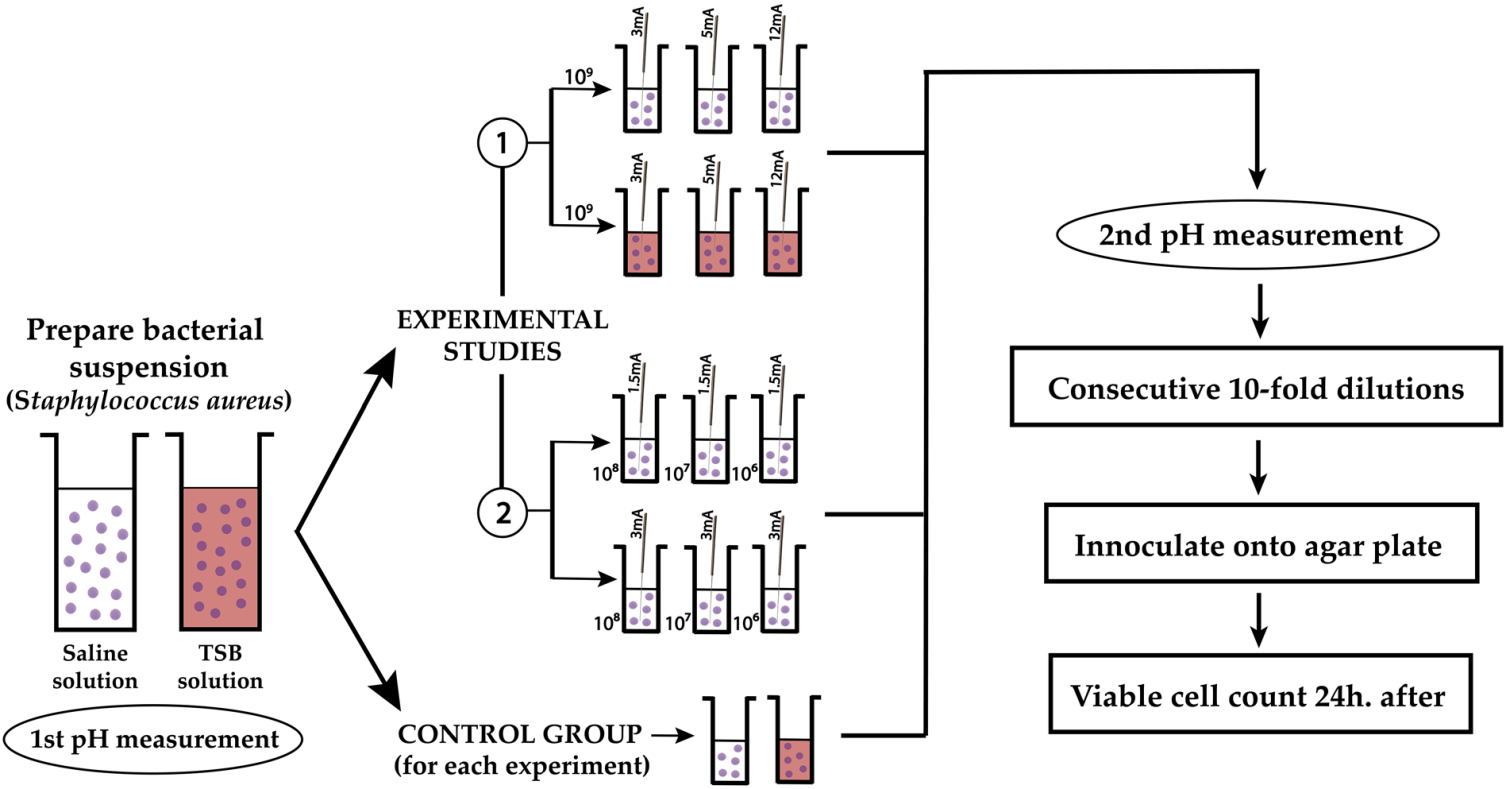
Flow-chart of the experimental design.

*Staphylococcus aureus* (ATCC 25923, purchased from Spanish Type Culture Collection) was cultured in Petri dishes (90 × 15 mm) containing Mueller–Hinton medium (bioMérieux, Spain) and incubated for 24 h at 37 °C. Several colonies (8–12 depending on size) were collected and transferred onto 5 mL of different media (Tryptic Soy Broth (TSB, bioMérieux, Spain)) or physiological saline solution (0.9% NaCl in water, Sigma-Aldrich, Spain). To standardize the number of bacteria in the suspension, we used a standard McFarland suspension of 4, which equals approximately 1.2 × 10^9^ Colony-Forming Units (CFU) per millilitre (or OD = 1 at 540 nm). From this initial suspension, decimal dilutions were made for use in the corresponding experiment (10^8^, 10^7^ and 10^6^ CFU/mL) (Fig. 1).

### Galvanic current source

The galvanic current generator used was Physio Invasiva (PRIM Physio, Móstoles, Spain). During all procedures, IPEN needles 0.32 × 30 mm (Agupunt, Barcelona, Spain) were used. Different intensities (from 1.5 to 12 mA) were used depending on the experiment.

**Figure 2:**
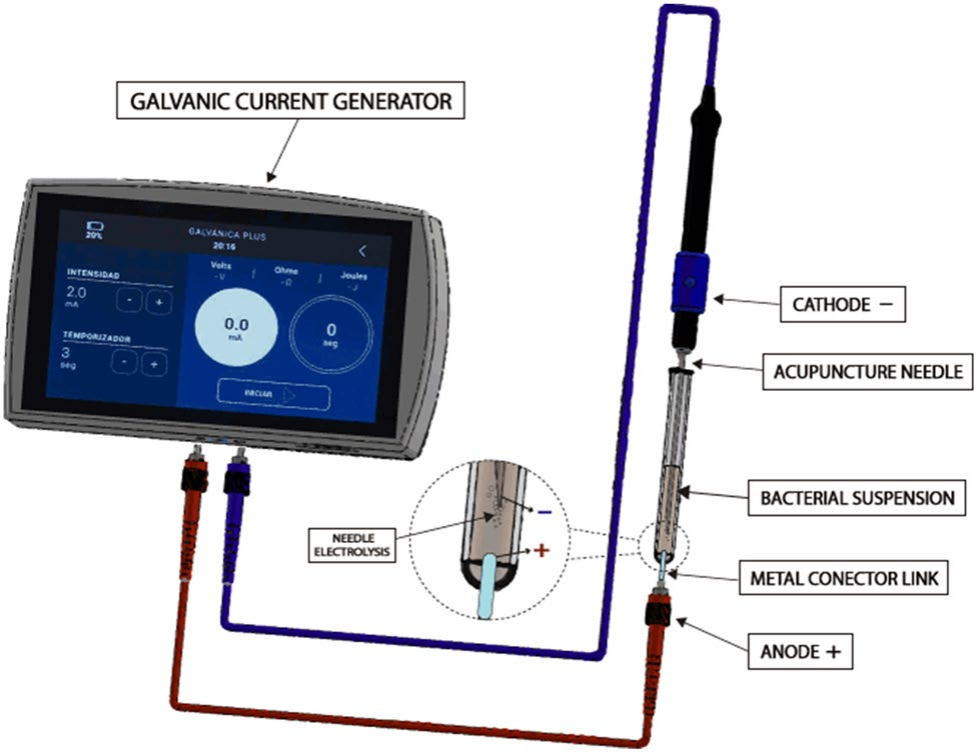
Procedure for PNE in vitro. Galvanic currents generate a cathodic flow in the bacterial suspension.

To simulate the clinical application of PNE, a biocompatible test tube material was used to serve as a container for the bacterial medium (Fig. 2). For each bacterial suspension, 150 μL per tube were placed before the treatments. At one end the anode was placed, coupled to the container through a conductor metal link^17^. On the other end the cathode was introduced (the needle 0.32 × 30 mm attached to the galvanic current generator).

### Experimental design

Two sequential experiments were conducted. The selection of variables for the second experiment was based on the results of the first, and based on usual clinical practice.

#### Experimental study 1

Three trials were conducted using a two-factor design with two independent variables (dose and type of solution) and two dependent variables (bacterial death levels and change in pH level). In each trial, in vitro subjects were randomly assigned to one of six groups determined by these two independent variables, receiving a combination of 3 or 5 or 12 mA (during 5 s and repeated 5 times) in saline or TSB media with a bacterial concentration of 1–1.4 × 10^9^ CFU/mL. A control group was created for each solution in each trial. Two dependent variables were measured: the change in the pH level, measured using a pH-meter GLP 21 (Crison Instruments S.A., Barcelona, Spain) and the reduction in the log CFU, in relation to the control group, which were measured at the end of each trial and 24 h after each trial, respectively.

Moreover, for this experiment we designed an additional and complementary experimental study involving only in the saline solution (the only one that lead to a significative reduction in the log CFU). This experiment was exclusively aimed to support statistical significance between certain doses of that type of solution (i.e. in order to build a narrower Confidence Interval (CI)). News in vitro subjects were generated with the same bacterial concentration and randomly assigned to one of those same doses (3 or 5 or 12 mA). A control group was also created.

#### Experimental study 2

An in vitro trial was conducted using a two-factor design with two independent variables (dose and bacterial concentration degree in the solution) and one dependent variable (bacterial death levels). Because the dose 3 mA showed significant reduction in the log CFU and that dose is the highest used in clinical practice for tendinopathies, this was the top dose used in this experiment. Likewise, high bacterial concentration is not usual in tendinopathies and other musculoskeletal disorders, we used lower bacterial concentrations (6, 7 and 8 Log_10_ CFU/mL) than experiment 1. In each trial, in vitro subjects were randomly assigned to one of six groups determined by these two independent variables, receiving a combination of 1.5 or 3 mA (during 5 s and repeated 5 times) in saline solution. A control group was created for each bacterial concentration in each trial.

### Bacteriological evaluation

Following all experiments, decimal serial dilutions (from 10^–1^ to 10^–7^) were prepared. Then, 100 μL of each dilution were seeded on the surface of Mueller–Hinton dishes in triplicate (bioMérieux) and incubated at 37 °C for 24 h (Fig. 1). The number of colonies was counted at the appropriate dilution and the number of CFU/mL were calculated.

### Statistical analysis

A two-way between-groups analysis of variance was conducted to firstly examine the impact of the dose and type of solution on levels of pH change and bacterial death (experimental study 1), and subsequently, to determine the impact of the dose and bacterial concentration degree on bacterial death levels (experimental study 2). Post-hoc comparisons were also employed with the Tukey HSD. When a disordinal interaction was present in these analyses, separate one-way ANOVAs were additionally used to explore the effect of the other variable. Also, one-way ANOVAs were calculated to explore the impact of the dose on bacterial death levels.

## Results

### Effect on bacterial death levels (from experimental studies 1 and 2)

**Figure 3:**
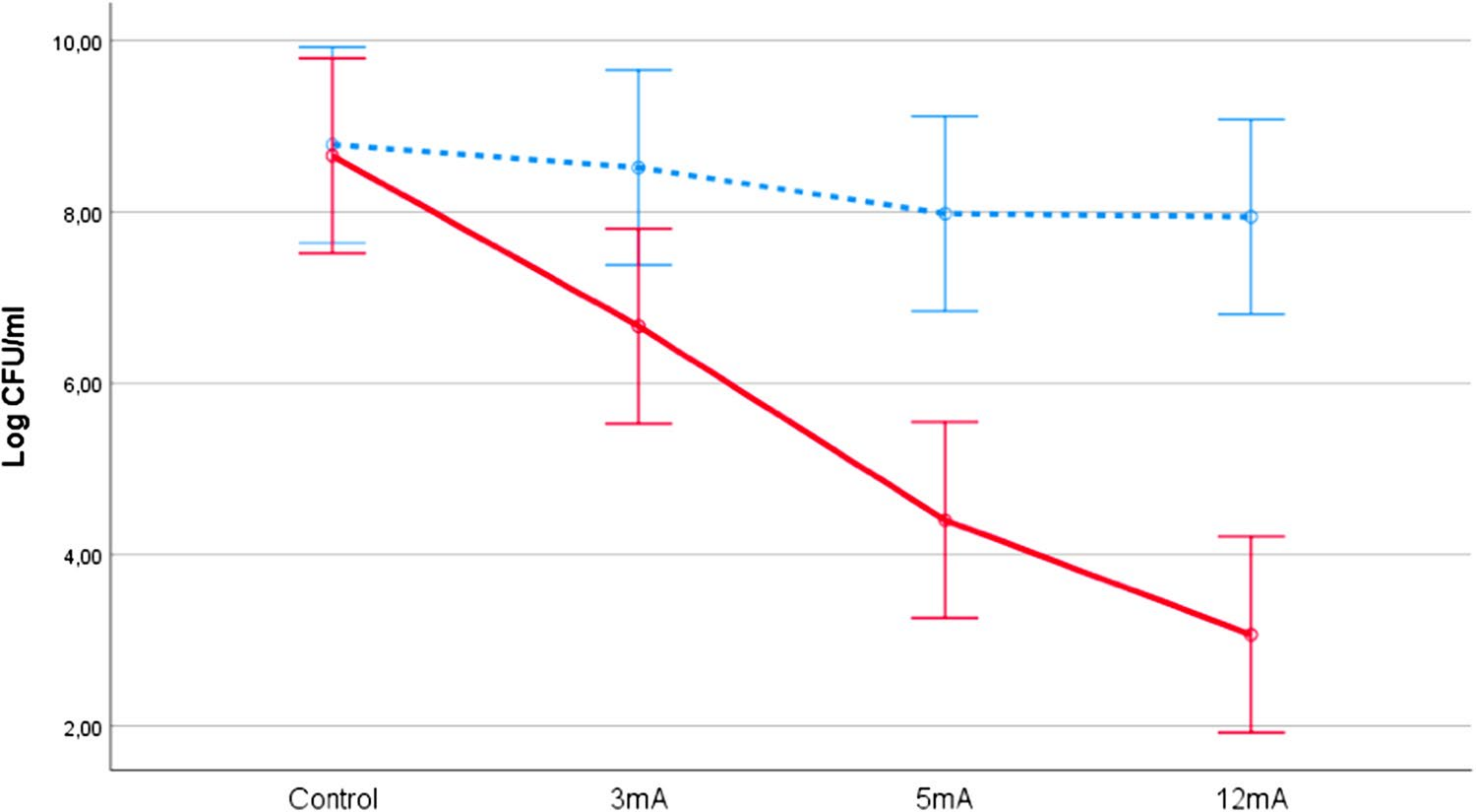
Effect of different doses of galvanic current on a 9 Log_10_ CFU/mL concentration of *S. aureus* in TSB (blue) and saline solution (red). All the doses were applied during 5 s and repeated 5 times. The error bars represent CI (95%).

Figure 3 shows that the relative effect of the saline solution was consistently higher than TSB bacterial solution at three-experimental doses and no significant interaction effect was observed between both types of solution and the three experimental doses (F(2,12) = 3.0, p = 0.09) on bacterial death levels. Although the targeted effect of the three-experimental doses showed a tendency towards an increase, their CIs overlapped in some cases (i.e., the effect of the 3 and 5 mA groups or between the 5 and 12 mA groups). Consequently, a complementary experimental study was conducted using saline solution.

**Figure 4:**
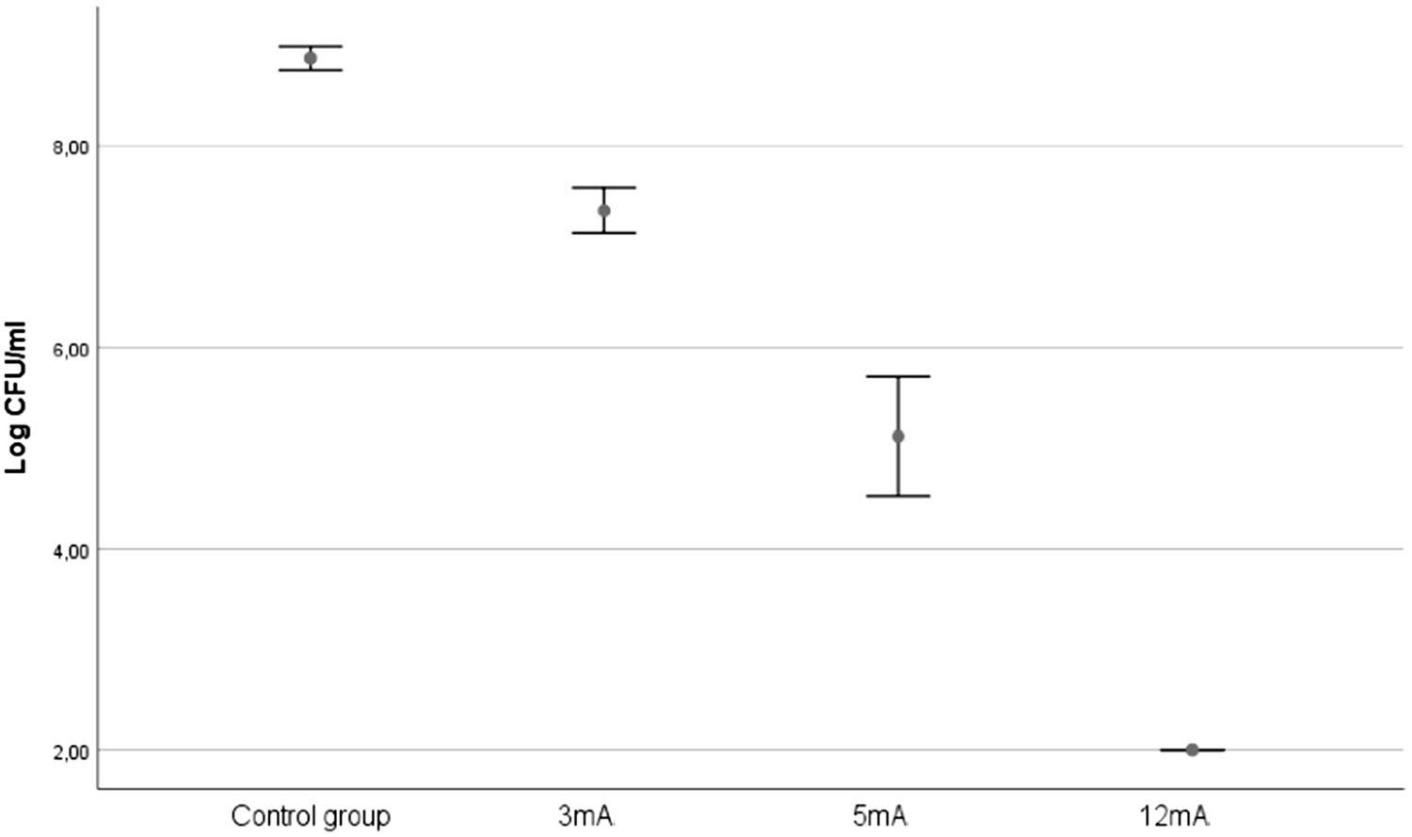
Complementary study of the effect of the application of different doses of galvanic current on a 9 Log_10_ CFU/mL concentration of *S. aureus* in saline solution. All the doses were applied during 5 s and repeated 5 times. The error bars represent the CI (95%).

Figure 4 shows the relative effect of the three experimental doses and control group in saline solution performed in a complementary experimental study. The means of each group were similar to those illustrated in Fig. 2, although the 95% CI were narrower. A Kruskal–Wallis test revealed a statistically significant difference in bacterial death levels across the four dose groups: H (3, 20) = 21.9, p < 0.001. Post-hoc comparisons indicated that all groups, including the 3 mA group (mean = 7.3; SD = 0.21), were significantly different at p < 0.001 in comparison to other groups.

**Figure 5:**
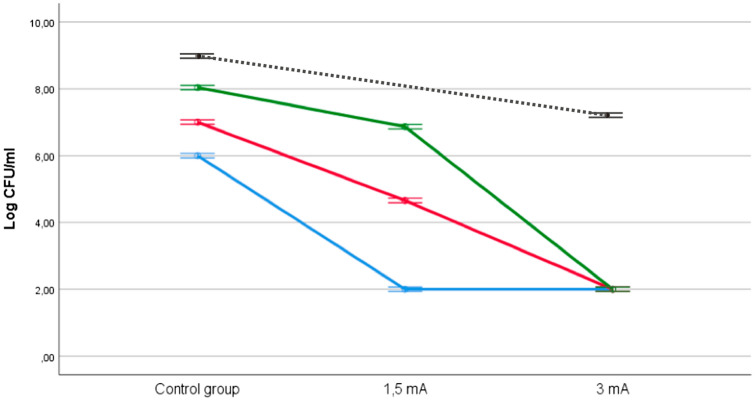
Effect of the application of a different dose of galvanic current in saline solution on three bacterial concentrations: 8 log_10_ CFU/mL (green), 7 log_10_ CFU/mL (red) and 6 log_10_ CFU/mL (blue). Dashed black line represent the reference data for 9 log_10_ CFU/mL using the data shown in Fig. 3. The error bars represent the CI (95%).

In relation to experimental study 2, Fig. 5 shows that, although the control groups from each of the three bacterial concentrations (i.e., 6, 7 and 8 Log_10_ CFU/mL) maintained a similar concentration, death levels of the two experimental doses were consistently higher at each these concentrations. Nevertheless, there was a significant interaction effect between the three types of concentration and two experimental doses (F(2,12) = 216, p < 0.001) on bacterial death levels. Figure 4 displays a disordinal interaction where the relative effect of the dose with 3 mA is consistently higher than the dose with 1.5 mA at levels of 8 log_10_ CFU/mL and 7 log_10_ CFU/mL, although not for the 6 log_10_ CFU/mL concentration.

An ANOVA test for the 1.5 mA group revealed a statistically significant difference between for the three concentration groups: F(2,6) = 216, p < 0.001). Post-hoc comparisons indicated that the mean score for group 8 log_10_ CFU/mL (mean = 6.8, SD = 0.1), 7 log_10_ CFU/mL (mean = 4.6, SD = 0.1) and 6 log_10_ CFU/mL (mean = 2, SD = 0) were significantly different among them. In contrast, no significant differences were found for the 3 mA group.

In both groups, 8 log_10_ CFU/mL and 7 log_10_ CFU/mL, t-tests showed significant differences at p < 0.001 in bacterial death levels between 1.5 mA and 3 mA (mean = 6.8, SD = 0.1 vs mean = 2, SD = 0, and mean = 4.6, SD = 0.1 vs mean = 2, SD = 0). In contrast, the group 6 log_10_ CFU/mL showed no statistically significant differences.

### Effects on the levels of increased pH (from experimental study 1)

**Figure 6:**
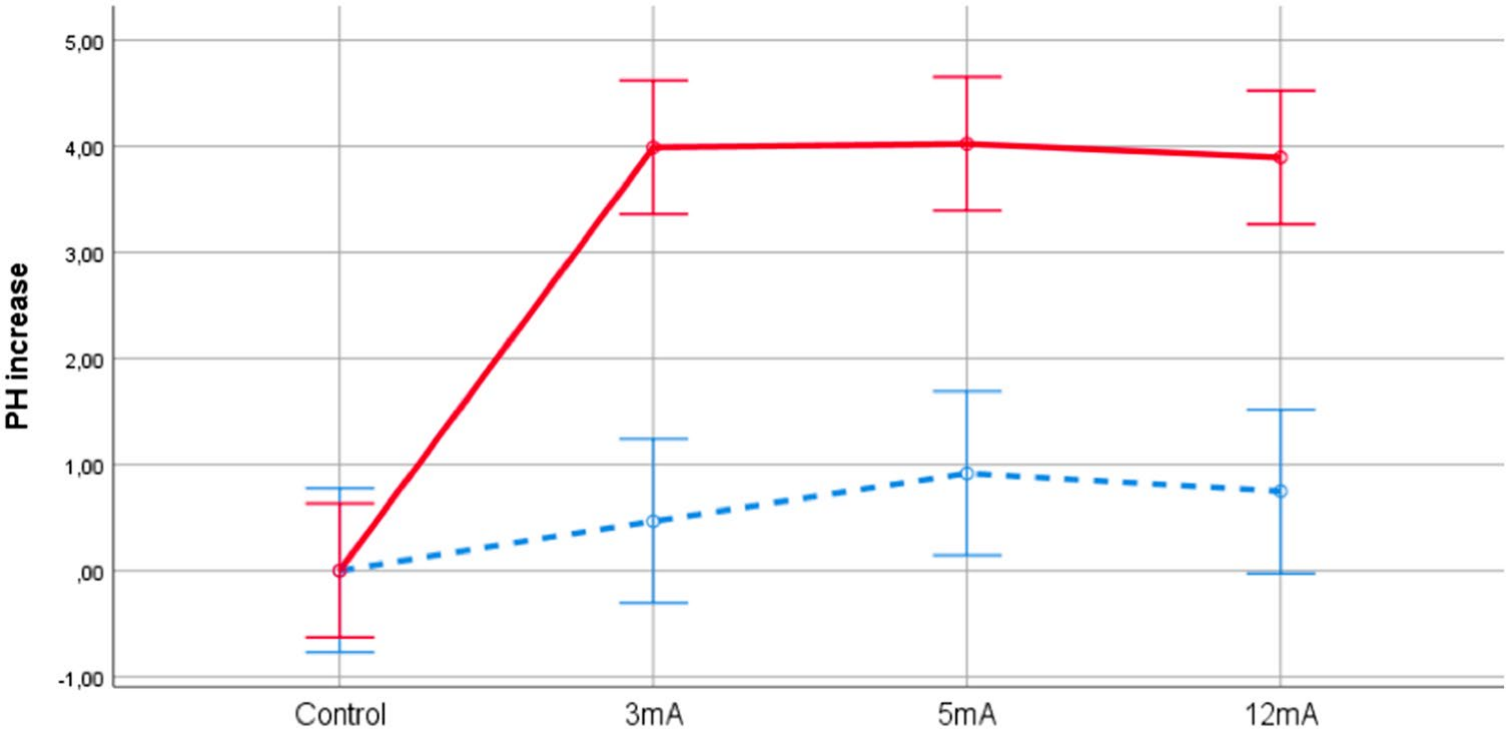
Effects of the application of different doses on the increase in pH in TSB (blue) and in saline solution (red). Baseline pH values had a mean of 6.04 (SD = 0.14) for TSB and 7.1 (SD = 0.07) for saline solution.

As displayed in Fig. 6, the relative effect of the saline solution (from a mean of 7.1 [SD = 0.01] at baseline to 11.19 [SD = 0.08] at the end) was consistently greater than TSB (from 7.1 [SD = 0.01] to 7.73 [SD = 0.17]) at three experimental doses, and this effect was significant compared to the control group. The results of the two-way Independent ANOVA revealed that there was not a significant interaction effect between the two types of solutions and the three experimental doses (F(2,9) = 0.189, p = 0.831) on increased pH levels. Within each solution, the levels of increased pH were similar between the three experimental doses, and thus the main effect for dose (F(2,9) = 0.213, p = 0.812) did not reach statistical significance. In contrast, there was a significant main effect for the type of solution (F(1,9) = 114, p < 0.001). The mean score for the saline solution across the three groups (mean = 4, SD = 0.5) was significantly higher than the mean TSB (mean = 0.5, SD = 0.5). The mean increase was 3.3 (95% CI 2.6–3.9).

## Discussion

To the best of our knowledge, this in vitro study is the first to evaluate the bactericidal effect of PNE. Our results revealed that the bactericidal effect of PNE depends on the solution where the bacteria are located. Furthermore, all the doses applied to saline solution, regardless of bacterial concentration level, generated a significant bacterial death of *S. aureus.* Thus, our results support the hypothesis postulated in previous research by authors such as Berna-Serna et al.^9^, who found that this technique was an effective option for the treatment of inflammatory pathologies with high levels of bacterial concentration, such as MF.

Aside from the demonstrated bactericidal power, our study has revealed that bacterial death levels depend on both the applied dosage and the bacterial concentration. Thus, the bacterial death levels recorded upon the highest bacterial concentration (9 log_10_ CFU/mL) were consistently increased with higher doses. However, we also found similar or higher death levels using lower doses in low bacterial concentrations (8, 7 and 6 log_10_ CFU/mL). For example, a dose of 0.075 Coulombs (i.e., resulting from 3 mA-5 s-5 times) applied to low concentrations achieved the same death level as the highest dose used upon the highest bacterial concentration. Nevertheless, the effect of the bacterial concentration on bacterial death levels did not consistently increase when doses were increased and we found a pattern of interaction between bacterial concentration and dosage. To date, no similar study exists, and therefore we are unable to compare our results with previous studies. Other authors such as Li et al.^18^ and Ciria et al.^19^ also used direct current, although at much higher doses (> 30 mA) and seeking the ablation effect on various types of tumors.

An unexpected result was the null bactericide effect of the TSB solution in all the doses employed. A possible explanation for this may be because TSB is a buffered medium, with molecules capable of partially neutralizing the hydroxyl radicals (–OH) generated in the cathode^20^ thus reducing the local effect of the technique.

Our findings regarding the power of PNE on low bacterial concentrations are highly relevant for clinical applications of this technique. Thus, these results may open a field of research in other skin pathologies or wounds with lower bacterial concentrations than MF. Furthermore, it supports the safety of the PNE technique although bearing in mind that the skin contains a microbiome with small amounts of bacterial strains such as *Staphylococcus*^7^ and the percutaneous application of the PNE increases the risk of dragging bacteria along the skin.

In addition, the finding that bacterial concentration interacts with dose has relevant applications. We found that the bactericidal effect for concentrations of 7 and 8 log_10_ CFU/mL increased with higher doses, however, the effect for the low concentration was the same in both doses used. Moreover, although the concentrations of 7 and 8 log_10_ CFU/mL had different effects using the lowest of two doses (1.5 mA), they had the same effect at the highest dose. Consequently, in clinical practice, the use of PNE for bacterial concentrations of 6 log_10_ CFU/mL or lower, a dose of 0.0375 Coulombs (i.e., resulting from 1.5 mA-5 s-5 times) may be enough to achieve the highest bacterial death level. For higher bacterial concentrations the use of higher doses may be more advisable.

Regarding pH changes, our study also found that change depends on the solution where the bacteria are located. The improved results in saline solution are consistent with a previous study^8^, employing aseptic solutions. Nevertheless, additionally, we found that the degree of pH change was constant when we increased the doses in both the saline and TSB solutions. Moreover, we verified that there was no relation between the levels of pH change and bacterial death with increasing dosage. In other words, both low and high doses are capable of generating the same basification of the solution, although they cause different levels of bactericidal effect.

Our study has several limitations. First, we only used the *S. aureus* and therefore further studies along this line of research are necessary to determine the effects of this type of galvanic current on other germs or bacterial strains. Second, in all experiments the time of application maintained constant and we changed the total dose by adjusting the intensity. Consequently, we are unable to determine to what extent the effects described depend on the intensity or the time of application. Likewise, further research is necessary using other types of solutions that more closely resemble human body fluid composition.

## Conclusions

In conclusion, PNE has a bactericidal effect against *S. aureus* and the level of this effect is mainly modulated by the solution, the bacterial concentration and the dose. Nevertheless, applications in saline solution with low bacterial concentrations generated almost total bacterial death, regardless of the applied dose. PH changes are modulated by solution regardless of the dose used. The mean pH of saline solution after electrolysis (11.19 [SD = 0.08]) was consistently greater than TSB (7.73 [SD = 0.17]). However, there is no relationship between the basification of the medium and the bactericidal effect.
